# A mitochondrial rRNA dimethyladenosine methyltransferase in Arabidopsis

**DOI:** 10.1111/j.1365-313X.2009.04079.x

**Published:** 2010-02

**Authors:** Uwe Richter, Kristina Kühn, Sachiko Okada, Axel Brennicke, Andreas Weihe, Thomas Börner

**Affiliations:** 1 Institut für Biologie/Genetik, Humboldt-UniversitätChausseestr. 117, 10115 Berlin, Germany; 2 Australian Research Council Centre of Excellence in Plant Energy Biology, University of Western AustraliaCrawley 6009, WA, Australia; 3 Molekulare Botanik, Universität Ulm89069 Ulm, Germany

**Keywords:** rRNA dimethyltransferases, mitochondria, Arabidopsis, mitochondrial transcription, molecular phylogeny

## Abstract

*S*-adenosyl-l-methionine-dependent rRNA dimethylases mediate the methylation of two conserved adenosines near the 3′ end of the rRNA in the small ribosomal subunits of bacteria, archaea and eukaryotes. Proteins related to this family of dimethylases play an essential role as transcription factors (mtTFBs) in fungal and animal mitochondria. Human mitochondrial rRNA is methylated and human mitochondria contain two related mtTFBs, one proposed to act as rRNA dimethylase, the other as transcription factor. The nuclear genome of *Arabidopsis thaliana* encodes three dimethylase/mtTFB-like proteins, one of which, Dim1B, is shown here to be imported into mitochondria. Transcription initiation by mitochondrial RNA polymerases appears not to be stimulated by Dim1B *in vitro*. In line with this finding, phylogenetic analyses revealed Dim1B to be more closely related to a group of eukaryotic non-mitochondrial rRNA dimethylases (Dim1s) than to fungal and animal mtTFBs. We found that Dim1B was capable of substituting the *E. coli* rRNA dimethylase activity of KsgA. Moreover, we observed methylation of the conserved adenines in the 18S rRNA of Arabidopsis mitochondria; this modification was not detectable in a mutant lacking Dim1B. These data provide evidence: (i) for rRNA methylation in Arabidopsis mitochondria; and (ii) that Dim1B is the enzyme catalyzing this process.

## Introduction

Mitochondria and plastids are descendants of bacterial endosymbionts (Margulis, 1970, 1981) and both possess their own vestigial genomes. Analyses of the mitochondrial DNA (mtDNA) trace the evolutionary predecessor of mitochondria to a single ancestor whose closest contemporary relatives are found within the α division of the proteobacteria (Yang *et al.*, 1985). The origin of chloroplasts traces back to a primary endosymbiotic event between a eukaryotic host and a relative of extant cyanobacteria representing the root of the plant kingdom (Rodriguez-Ezpeleta *et al.*, 2005). The majority of the original set of mitochondrial and plastid genes was either relocated to the nuclear genome or lost from the cell relatively early in the process of both endosymbiotic events (Gray *et al.*, 1999). As a consequence of the unidirectional functional gene transfer, components participating in the diverse mitochondrial and plastid metabolic pathways and genetic processes are largely encoded in the nucleus and, following synthesis in the cytosol, are imported into the organelles (Peeters and Small, 2001; Herrmann, 2003).

Surprisingly, several mitochondrial DNA replication genes were acquired probably from a T-odd phage early in the evolution of the eukaryotic cell, at the time of the mitochondrial endosymbiosis (Shutt and Gray, 2006a). Similarly, in mitochondria of the budding yeast *Saccharomyces cerevisiae* (Greenleaf *et al.*, 1986; Masters *et al.*, 1987), mammals (Tiranti *et al.*, 1997; Gaspari *et al.*, 2004), plants (Weihe *et al.*, 1997) and other eukaryotes (Cermakian *et al.*, 1996), a nucleus-encoded T-odd phage-type RNA polymerase (RNAP) has replaced the ancestral bacterial-type RNAP.

Unlike the single-subunit RNA polymerases of bacteriophages, mitochondrial phage-type RNA polymerases require auxiliary factors to initiate transcription at promoter sequences. To date, two types of nucleus-encoded mitochondrial transcription factors, designated here as mtTFA and mtTFB, have been characterized in *S. cerevisiae* (Winkley *et al.*, 1985; Schinkel *et al.*, 1987a; Matsunaga and Jaehning, 2004), *X. laevis* (Bogenhagen and Insdorf, 1988; Bogenhagen, 1996), *Drosophila melanogaster* (Matsushima *et al.*, 2004, 2005), humans (Fisher and Clayton, 1988; Falkenberg *et al.*, 2002; McCulloch *et al.*, 2002) and mouse (Parisi and Clayton, 1991; Gaspari *et al.*, 2004). The HMG-box protein mtTFA is an essential transcription factor in human (Gaspari *et al.*, 2004), but not in yeast mitochondria where it is an abundant DNA-binding protein that enhances transcriptional activity (Parisi *et al.*, 1993; Kanki *et al.*, 2004).

In contrast, mtTFBs belonging to the family of *S*-adenosyl-l-methionine (SAM)-dependent rRNA adenine dimethyltransferases were found to be essential for transcription in yeast and mammalian mitochondria (Jang and Jaehning, 1991; Asin-Cayuela and Gustafsson, 2007). Homologues of this rRNA adenine dimethylase protein family are found to function in all domains of life. SAM-dependent rRNA dimethylases, such as KsgA from *E. coli* or the nucleolar/cytoplasmic Dim1 of *S. cerevisiae*, usually mediate the name-giving methylation activity and modify two specific adenosines in a highly conserved stem-loop near the 3′ end of small subunit ribosomal RNAs. While the significance of this methylation activity is poorly understood, it seems that KsgA/Dim1 orthologues may have the general potential to play diverse additional roles within different compartments of the cell (Lafontaine *et al.*, 1995, 1998; Tokuhisa *et al.*, 1998; Metodiev *et al.*, 2009). For the yeast protein ScDim1 it was shown that the enzymatic cytoplasmic function of Dim1 in dimethylation can be separated from an involvement in pre-rRNA processing (Lafontaine *et al.*, 1998).

Mitochondria of all metazoan species investigated thus far possess two mtTFB proteins. In contrast, mitochondria of *S. cerevisiae* and other fungal species possess only one mtTFB. Yeast sc-mtTFB lacks rRNA dimethyltransferase activity correlated with the lack of the corresponding modification in the mitochondrial rRNA of budding yeast (Cotney and Shadel, 2006). Recent observations led to the conclusion that despite the similarity of both proteins to rRNA dimethyltransferases and their ability to act as transcription factors *in vitro*, the methyltransferase and cofactor roles are distributed *in vivo* between TFB1M (methyltransferase) and TFB2M (transcription factor) in animal mitochondria (Falkenberg *et al.*, 2002; McCulloch and Shadel, 2003; Matsushima *et al.*, 2004, 2005; Cotney *et al.*, 2007; Metodiev *et al.*, 2009).

In dicotyledonous plants such as *Arabidopsis thaliana*, three phage-type RNAPs are involved in the transcription of organellar genes: a mitochondrial RNAP (RpoTm), a plastid RNAP (RpoTp) and an RNAP dual-targeted to both mitochondria and plastids (RpoTmp; Hedtke *et al.*, 1997, 2000). Previous studies suggested that factors supporting mitochondrial RNAPs in promoter recognition and/or transcription initiation should exist also in higher plants (Newton *et al.*, 1995; Young and Lonsdale, 1997; Binder and Brennicke, 2003). More recently we found that Arabidopsis RpoTm and RpoTp are able to correctly start transcription *in vitro* from several mitochondrial and at least one plastid promoter without the assistance of further protein factors. Selective promoter recognition by RpoTm and RpoTp and the apparent inability of RpoTmp to correctly initiate transcription at mitochondrial and plastidial NEP promoters imply, however, that auxiliary factors are required for efficient initiation of transcription *in vivo* (Kühn *et al.*, 2007).

Only limited information is available about mitochondrial rRNA methylation in the green branch of life (Schnare and Gray, 1982) and so far mtTFA- and mtTFB-related transcription factor proteins have not been found in plants. We therefore initiated this study to ascertain if the Arabidopsis mitochondrial rRNA shows the conserved type of adenine dimethylation and if plant mitochondria have one or more dimethylases belonging to the KsgA/Dim1 family. Moreover, we were interested in the potential function of such a methyltransferase as a mitochondrial transcription factor.

We report here on KsgA/Dim1-like Arabidopsis proteins initially identified by in-silico screening of databases. One of these proteins is the Pfc1 previously demonstrated to function as a plastidial 16S rRNA dimethylase (Tokuhisa *et al.*, 1998). The two others are shown here to be localized to the nucleus and to mitochondria, respectively. We found no evidence for an mtTFB-like role of the mitochondrial protein, designated as Dim1B (adenosine dimethyl transferase 1B), in mitochondrial transcription. Our data demonstrate, however, that Dim1B is an rRNA methyltransferase and is required for the dimethylation of two conserved adenines in the mitochondrial 18S rRNA of Arabidopsis.

## Results

### mtTFB/rRNA dimethyltransferase-like proteins in Arabidopsis

To screen the Arabidopsis genome for sequences encoding mtTFB-like proteins we queried the genome and EST databases with the putative *Schizosaccharomyces pombe* mtTFB amino acid sequence (McCulloch *et al.*, 2002) and the human mtTFB1 and mtTFB2 sequences (Falkenberg *et al.*, 2002) using the BLASTP and TBLASTN algorithms available at the National Centre for Biotechnology Information (NCBI). The search predicted three mtTFB/rRNA dimethyltransferase-like proteins to be encoded by the loci At5g66360, At2g47420 and At1g01860 in Arabidopsis. At1g01860 corresponds to the previously characterized *Pfc1* gene coding for a plastidial 16S rRNA dimethylase (Tokuhisa *et al.*, 1998).

The putative rRNA dimethyltransferase genes at loci At2g47420 and At5g66360 were tentatively designated *Dim1A* and *Dim1B*, respectively. *Dim1A* is predicted to encode a 353-amino acid polypeptide. Available EST data support alternative splicing of the hypothetical mRNA deriving from *Dim1B*, which would give rise to two different polypeptides of 352 and 380 amino acids (Accession numbers. NP_201437 and NP_975003). However, PCR amplification of the *Dim1B* coding sequence from cDNA yielded only the splice product coding for the longer of the two predicted products. For *Dim1A*, a cDNA sequence of the expected length was amplified (data not shown). Protein sequence comparisons revealed that the annotated optional *Dim1B* intron codes for amino acid sequence motifs that are conserved among rRNA dimethylase-like proteins (amino acids 214–241 of the derived Dim1B polypeptide). Hence, further sequence analyses were based on the longer deduced Dim1B polypeptide. Both Dim1A and Dim1B display ∼27% and ∼30% amino acid sequence similarity to mtTFB from *S. pombe* and *S. cerevisiae*, respectively; ∼12% and ∼15% of the predicted amino acids are identical when sequences exclusive of the predicted transit peptides are compared. These levels of similarity approximately correspond to those between human and fungal mtTFB sequences. Similarities of Dim1A and Dim1B with each human TFB1M and TFB2M are ∼37%; amino acids are for both Dim1A and Dim1B ∼20% identical with the human sequences.

### Subcellular localization of the mtTFB-like proteins Dim1A and Dim1B

**Figure 1:**
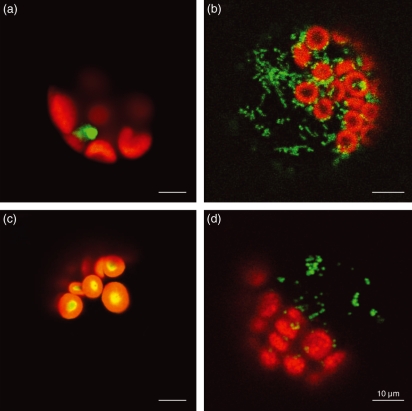
Transient expression of GFP fusion proteins in *Arabidopsis thaliana* protoplasts. The *Dim1A* and *Dim1B* gene fragments encoding putative transit peptides were inserted into plasmid pOL–GFPS65C to generate vectors driving the expression of Dim1A–GFP (a) and Dim1B–GFP (b) showing nuclear (a) and mitochondrial (b) GFP localisation. Control constructs code for plastidial RecA–GFP (c) and mitochondrial CoxIV–GFP (d), respectively. Images were taken by confocal fluorescence microscopy.

Several computer algorithms consistently predict Dim1B to possess an N-terminal transit peptide mediating the import of the protein into mitochondria while Dim1A might be a nuclear protein (Table S1). To experimentally investigate the subcellular localization of Dim1A and Dim1B, nucleotide sequences encoding the full length and 64 N-terminal amino acids of Dim1A and Dim1B, respectively, were fused in-frame to the green fluorescent protein (GFP) coding sequence. Arabidopsis protoplasts were transformed with the Dim1A– and Dim1B–GFP fusion constructs and transient expression of the fusion proteins was monitored using confocal laser fluorescence microscopy. Two plasmids encoding a mitochondrial Cox4–GFP or a plastidial RecA–GFP fusion protein were used for reference transformations of Arabidopsis protoplasts. Protoplasts expressing Dim1B–GFP displayed green fluorescence of small structures resembling the fluorescent mitochondria of protoplasts synthesizing Cox4–GFP (Figure 1), substantiating a mitochondrial localization of Dim1B. Dim1A–GFP fluorescence, on the other hand, was found to localize to the nucleus and for the most part enveloped a small globular structure therein, which likely corresponds to the nucleolus. These data indicate that Dim1A does not possess a mitochondrial or plastidial transit peptide.

### Phylogenetic analysis of plant, fungal and animal rRNA dimethylase-like proteins

**Figure 2:**
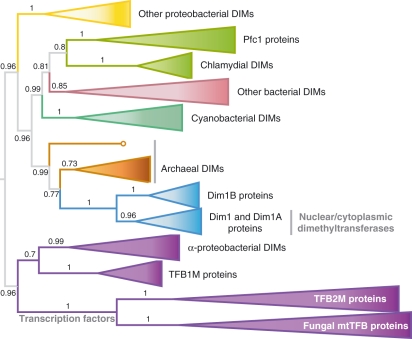
Phylogeny of mitochondrial transcription factors and small-subunit rRNA dimethylases. Bayesian phylogenetic analysis from conserved amino acid sequence sections indicated in the amino acid sequence alignment (see Figure S1). Collapsed branches of orthologous and paralogous proteins from the detailed phylogenetic tree (see Figure S2) are displayed as triangles. Total branch length to the closest and the farthest leaf are used to calculate the length of the triangles.

In order to assess the phylogenetic relationships of Arabidopsis Dim1A, Dim1B and of the plastidial methyltransferase Pfc1 to established mitochondrial transcription factors such as yeast and animal mtTFBs and to other rRNA dimethylases such as *E.* *coli* KsgA, the Dim1A, Dim1B and Pfc1 sequences were compared with available mtTFB sequences and to a set of sequences of characterized and predicted rRNA dimethylases. Blast analyses with *Arabidopsis thaliana* Dim1A and Dim1B of all plant genomes available at the Joint Genome Initiative database (http://genome.jgi-psf.org/) including *Cyanidioschyzon merolae*, *Chlorella vulgaris*, *Ostreococcus tauri*, *Ostreococcus lucimarinus*, *Volvox carteri*, *Chlamydomonas reinhardtii*, *Physcomitrella patens* and *Selaginella moellendorffii* combined with searches against the NCBI Plant EST database revealed that Dim1B-like proteins might be restricted to flowering plants. For identified plant Dim proteins, subcellular targeting was predicted employing the *TargetP*, *Predotar*, *iPsort* and *MultiLoc* algorithms (Table 1). All sequences with high similarity to Arabidopsis Dim1B, which are therefore designated Dim1B in the phylogenetic tree, were predicted to contain putative mitochondrial transit peptides. Proteins most similar to Arabidopsis Dim1A were predicted to be neither plastidial nor mitochondrial (Table 1) and are referred to as Dim1A. Notably, the *Physcomitrella* genome was found to lack a Dim1B homologue but encodes two potential dimethyltransferases which best align with plant Dim1A proteins, PpDim1A-1 being predicted to be neither mitochondrial nor plastidial and PpDim1A-2 containing a putative mitochondrial transit peptide (Table 1). Amino acid sequences were compared using the Multalin algorithm (Corpet, 1988) and the alignment was refined according to a structure-based alignment generated for fungal mtTFBs and the *Bacillus subtilis* rRNA dimethylase ErmC’ (Schubot *et al.*, 2001; see Figure S1). Based on the selected sequence blocks indicated in the alignment, a Bayesian phylogenetic tree was derived (Figures 2 and S2). Essentially the same tree topology was obtained employing neighbour joining (NJ) or maximum likelyhood (ML) analysis.

**Table 1:** Prediction of subcellular localisations for Dim1A, Dim1B and Pfc-like proteins

	Predotar^a^	iPSORT^b^	TargetP^c^	MultiLoc^d^
CrPfc	Other	mt	mt/pt	pt
VcPfc	mt	mt	mt/pt	pt
OtPfc	mt/pt	mt	mt/pt	pt
PpPfc	pt	pt	pt	pt
SmPfc	mt	pt	pt	pt
PgPfc	pt	pt	pt	pt
Magnoliophyta Pfc	pt	pt	pt	pt
Magnoliophyta Dim1A	NOR (mt,pt)	NOR (mt,pt)	NOR (mt,pt)	nc or cyt
Magnoliophyta Dim1B	mt	mt	mt	mt
PpDim1A-2	mt	mt	mt	mt

Predictions of targeting to the mitochondrion (mt), nucleus (nc), cytoplasm (cyt) or plastid (pt) by the respective computer algorithms are documented. Proteins are designated as follows: CrPfc, *Chlamydomonas reinhardtii* Pfc; VcPfc, *Volvox carteri* Pfc; OtPfc, *Ostreococcus tauri* Pfc; PpPfc, *Physcomitrella patens* Pfc; SmPfc, *Selaginella moellendorffii* Pfc; PgPfc, *Pinus glauca* Pfc; PpDim1A-2, *Physcomitrella patens* Dim1A-2. Magnoliophyta Dim1A and Dim1B include predictions for proteins from *Arabidopsis thaliana*, *Vitis vinifera*, *Medicago truncatula*, *Lycopersicon esculentum*, *Populus trichocarpa*, *Zea mays* and *Oryza sativa*.

a http://urgi.versailles.inra.fr/predotar/predotar.html.

b http://hc.ims.u-tokyo.ac.jp/iPSORT/.

c http://www.cbs.dtu.dk/services/TargetP/.

d http://www-bs.informatik.uni-tuebingen.de/Services/MultiLoc.

Arabidopsis Dim1A and its plant orthologues appear to be most closely related to a group of rRNA dimethylases containing the yeast nucleolar 18S rRNA dimethylase Dim1. Therefore, they most probably represent nuclear/cytoplasmic enzymes, a suggestion which would be consistent with computational predictions of the subcellular localization of these proteins and with our GFP import experiments conducted with Arabidopsis Dim1A. A sister group to plant, fungal and animal nuclear Dim1-like dimethylases is formed by the predicted mitochondrial rRNA dimethylases of plants including Arabidopsis Dim1B. Remarkably, amino acid sequence similarities of Arabidopsis Dim1A and Dim1B to yeast Dim1 are 68% and 54%, respectively and considerably exceed similarities to yeast or animal mtTFBs. This is contrasted by similarities of yeast mtTFB, human TFB1M and TFB2M to yeast Dim1 with only 36%, 35% and 28%. The phylogram shows that the presumed plant mitochondrial rRNA dimethylases clustering with either Dim1A or Dim1B are decidedly more closely related to nuclear/cytoplasmic enzymes of this type – notably with the archaeal Dim1-like proteins at the root of this clade – than to fungal and animal mtTFBs.

On the other hand, the mtTFB sequences form a common branch with alpha-proteobacterial dimethyltransferases as previously described (Shutt and Gray, 2006b). Arabidopsis Pfc1 and its plant orthologues compose a distinct group apart from the Dim1A/Dim1B cluster and from three other well separated groups formed by animal TFB1Ms, animal TFB2Ms and the highly diverse fungal mtTFBs. In agreement with a recent study (Park *et al.*, 2009), the Pfc1-like sequences, which likely code for plastidial dimethyltransferases, unexpectedly form a common clade with chlamydial sequences but not with cyanobacterial or mitochondrial dimethyltransferases (Figures 2 and S2).

Given the presence of apparently only one mtTFB/rRNA dimethylase-like protein in plant mitochondria, our further experiments were designed to answer the question of whether Arabidopsis Dim1B could play a dual role as a mitochondrial rRNA dimethyltransferase and as a mitochondrial transcription factor.

### Promoter-specific transcription initiation is not stimulated by Dim1B

Recombinant Arabidopsis Dim1B was prepared in order to assay the protein *in vitro* for a possible function as cofactor in mitochondrial transcription. Dim1B fused to an N-terminal hexahistidine tag was expressed in *E. coli* and soluble recombinant protein was enriched from bacterial extracts; Dim1A was similarly prepared so as to have a non-mitochondrial rRNA dimethylase-like protein available for control experiments (Figure S3). In order to confirm the correct folding and native conformation of the recombinant proteins, electrophoretic mobility shift assays of Dim1A and Dim1B were carried out with mitochondrial DNA probes following the protocol described for testing DNA binding by human TFB1M (McCulloch *et al.*, 2002). Both Arabidopsis proteins were found to bind to DNA without showing sequence specificity (Figure S3).

It has been demonstrated that the yeast mitochondrial phage-type RNA polymerase RPO41, which on its own transcribes double-stranded linear DNA only non-specifically, recognizes mitochondrial promoters on a linear DNA template when complemented with sc-mtTFB in *in vitro* transcription experiments (Schinkel *et al.*, 1987b). We therefore used our previously established *in vitro* transcription system (Kühn *et al.*, 2007) to test if recombinant Dim1B enabled the Arabidopsis mitochondrial RNA polymerases RPOTm or RPOTmp to specifically transcribe from mitochondrial promoters located on linear DNA templates. We also examined if, in the presence of Dim1B, the two RNA polymerases recognized promoters on both linear and supercoiled DNA that in our earlier study (Kühn *et al.*, 2007) were not specifically utilized by any of the two enzymes.

Recombinant RPOTm and RPOTmp were assayed for transcription initiation at several mitochondrial promoters (promoter sequences are listed in Table S2) in the presence or absence of equimolar amounts of Dim1B; in order to discern non-specific effects of Dim1B addition, control reactions were set up with Dim1A (Figure S4). Figure S4 shows the PAGE analysis of transcripts synthesized *in vitro* from linear or supercoiled DNA templates containing the mitochondrial promoters P*atp6-1*-156 and P*atp6-1*-200, P*atp8*-157 and P*atp8*-228/226, or P*rrn18*-69 and P*rrn18*-156. RPOTm did not specifically transcribe from any mitochondrial promoter on a linear DNA template in the presence or absence of Dim1B; neither did the enzyme show increased mitochondrial promoter specificity due to Dim1B addition when supercoiled templates were provided. None of the promoters not utilized by RPOTm in the absence of Dim1B, like P*atp6-1*-156 or P*rrn18*-69, was recognized when Dim1B was added. The transcriptional activity and initiation specificity of RPOTmp were similarly unaltered by the addition of Dim1B; no significant promoter specificity of this enzyme was seen in the presence or absence of Dim1B with any mitochondrial promoter tested (only experiments with supercoiled templates are shown for RPOTmp). In addition to the three DNA templates analysed as shown in Figure S4, several other DNA templates with different promoters were tested. Promoter recognition assays are summarized in Table 2. Altogether, the addition of Dim1B appears to have no effect on RPOTm- or RPOTmp-driven transcription.

**Table 2:** Promoter recognition by RPOTm *in vitro*

		Promoter recognition RPOTm				Promoter recognition RPOTmp			
*In vitro* transcripti on template		ccc template		Linear template		ccc template		Linear template	
Plasmid	Promoters	−Dim1B	+Dim1B	−Dim1B	+Dim1B	−Dim1B	+Dim1B	−Dim1B	+Dim1B
pKL23-*atp6-1*	P*atp6-1*-156	−	−	−	−	−	−	−	−
	P*atp6-1*-200	+	+	−	−	−	−	−	−
pKL23-*atp6-1*-B	P*atp6-1*-916/913	+	+	na	na	−	−	na	na
pKL23-*atp6-2*	P*atp6-2*-436	+	+	na	na	−	−	na	na
	P*atp6-2*-507	+	+	na	na	−	−	na	na
pKL23-*atp8*	P*atp8*-157	−	−	−	−	−	−	na	na
	P*atp8*-228/226	+	+	−	−	−	−	na	na
pKL23-*atp9-*B	P*atp9*-239	−	−	−	−	−	−	na	na
	P*atp9*-295	+	+	−	−	−	−	na	na
pKL23-*atp9*	P*atp9*-487	+	+	na	na	−	−	na	na
	P*atp9*-652	+	+	na	na	−	−	na	na
pKL23-*cox2*	P*cox2*-210	+	+	−	−	−	−	na	na
	P*cox2*-481	+	+	−	−	−	−	na	na
pKL23-*rps3*	P*rps3*-1053	−	−	−	−	−	−	−	−
	P*rps3*-1133	−	−	−	−	−	−	−	−
pKL23-*rrn18*	P*rrn18*-69	−	−	−	−	−	−	na	ma
	P*rrn18*-156	+	+	−	−	−	−	na	na
pKL23-*rrn26*	P*rrn26*-893	+	+	−	−	−	−	na	na
pKL23-*trnM*	P*trnM*-98	+	+	−	−	−	−	na	na
pKL23-*trnM*-B	P*trnM*-574/573	−	−	na	na	−	−	na	na

### Dim1B is an rRNA dimethyltransferase

**Figure 3:**
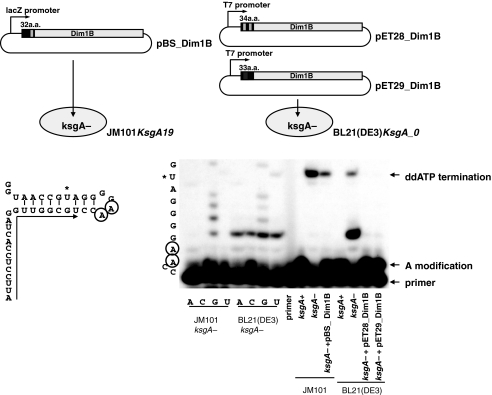
The *Dim1B* gene from *Arabidopsis thaliana* can complement site specific methylation defects in *E. coli*. The coding sequence of the *Dim1B* gene from *Arabidopsis thaliana* was cloned into vectors driving transcription in *E. coli* from either a lacZ or a T7 promoter as indicated in the scheme and introduced into the *E. coli* strains JM101 or BL21(DE3) lacking the *ksgA* gene. The specific adenosine dimethylation in the 3′ terminal stem–loop structure of the bacterial 16S rRNA is depicted on the left. Primer extension in the presence of ddATP confirms the absence of this methylation in the ksgA− mutant bacteria and yields no signal at the adenosine doublet but terminates primer extension at the next uridine in the 16S rRNA sequence. Introduction of a functional *ksgA* gene or either of the expression constructs of the *Dim1B* gene from Arabidopsis restores the methylation signal at the adenosine dinucleotide. The left part of the gel image shows the corresponding RNA sequence reactions for orientation of the ddATP termination signals.

To determine if the Dim1B protein is a functional rRNA dimethyltransferase, the complementation of an *E. coli* mutant lacking the N6-adenine dimethyltransferase encoded by the *KsgA* gene (*KsgA*^−^*E. coli*) was investigated. The bacterial strain was assayed for complementation by the introduced *Dim1B* gene from Arabidopsis. This strategy essentially follows the complementation assays as described by Seidel-Rogol *et al.* (2003) for complementing KsgA-deficient *E. coli* with human TFB1M. N6-adenine methyltransferase activity in *KsgA*^*−*^*E. coli* was recovered when the Arabidopsis *Dim1B* gene was introduced (Figure 3), indicating that Dim1B indeed is a functional and site-specific rRNA dimethylase. In both expression systems used, with the *Dim1B* gene activated either from the LacZ promoter (JM101) or from the T7 promoter (BL21(DE3)), N6-adenine methyltransferase activity was observed. In the LacZ system, the methylating enzyme activity seems to be weaker than in the T7 system, which may be caused by the weaker promoter and the resulting lower level of protein expression. In the T7 system, an extra primer extension product terminating at G_1517_ is observed. In the bacterial small ribosomal rRNAs such an m^2^G modification at position G_1516_ has been reported. However, the correspondence of this extension product with this modification needs to be confirmed by further analysis.

These complementation assays show that the Dim1B protein encoded by the *Dim1B* gene is an active N6-adenine methyltransferase which modifies the AA dinucleotide at the bacterial 16S rRNA nucleotides A1518 and A1519, the positions equivalent to the plant mitochondrial 18S rRNA nucleotides A1914 and A1915. To analyse the functional importance of this modification, the requirement of the *Dim1B* gene for plant growth was investigated.

### A *dim1B* mutant lacks adenosine modification of the mitochondrial 18S rRNA

**Figure 4:**
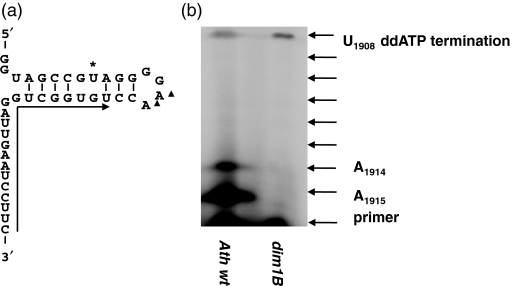
Arabidopsis *dim1B* plants lack adenosine modification of the mitochondrial 18S rRNA. (a) Sequence and predicted structure of the 3′ end of the mitochondrial small subunit 18S rRNA, the arrow indicating the reverse complementary primer used in the assay. (b) Poisoned primer extension products using wild-type (wt) and *Arabidopsis thaliana dim1B* plants (Δ*Atdim1B*) in which the *Dim1B* gene is disrupted. In wt plants ddATP incorporation into the cDNA strand at the methylated adenosine doublet results in a strong termination signal at these nucleotides and only a weak read-through signal at the next uridine. In the *dim1B* mutant no termination is detected at the adenosines and the read-through signal is increased.

A mutant line of Arabidopsis (GABI-Kat 071F10) that contains a T-DNA fragment inserted in the second exon of the *Dim1B* gene (see Experimental Procedures) and designated as *dim1B* was employed to analyse the role of Dim1B in mitochondrial RNA metabolism. The mutation had no apparent effect on plant growth or development under standard growth conditions (data not shown). As reverse transcriptase is unable to extend a DNA oligonucleotide primer if it encounters an adenosine dimethylated at the N-6 position of the base (Hagenbüchle *et al.*, 1978; Sigmund *et al.*, 1988), we used primer extension to distinguish between the modification states of wild-type and *dim1B* rRNAs. As shown in Figure 4, primer extension was inhibited at positions A1914/A1915 suggesting modification of the mitochondrial 18S rRNA at these nucleotides in the wild type. This signal was absent in RNA from the homozygous insertion line *dim1B*, where reverse transcription did continue uninhibited to position U1908. These results indicate that the conserved modification of two adenosines at the 3′ end of the ssrRNA is present in the 18S rRNA of Arabidopsis wild-type mitochondria and that *dim1B* plants lack this methylation, confirming that Dim1B is essential for methylation of the adenosine dinucleotide.

## Discussion

To investigate their potential function in mitochondrial transcription initiation and/or as bona fide dimethyltransferases, we screened the Arabidopsis genome and plant EST databases for genes related to members of a gene family encoding rRNA dimethyltransferases, some of which act as transcription factors in fungal and animal mitochondria. These screens revealed two uncharacterized genes encoding dimethylase-like proteins, *Dim1A* (At2g47420) and *Dim1B* (At5g66360), in addition to *Pfc1* (At1g01860) previously characterized as the gene coding for a plastidial 16S rRNA adenosine dimethylase (Tokuhisa *et al.*, 1998). The results of the present study strongly suggest that *Dim1A* encodes a nuclear and *Dim1B* encodes a mitochondrial rRNA dimethyltransferase. The protein structure and the observed non-specific DNA binding activities of Dim1A and Dim1B are compatible with a functional role of both proteins as rRNA dimethyltransferases, but had also been observed with mtTFBs (Riemen and Michaelis, 1993; McCulloch *et al.*, 2002).

The expression of Dim1A fused to GFP in Arabidopsis protoplasts led to the appearance of GFP fluorescence within the nucleus where it localized to structures probably representing nucleoli. Such a localization of Dim1A is in agreement with predictions based on properties of the N-terminal domain of this protein. Moreover, it would also fit a role of Dim1A as an enzyme that interacts with pre-90S during early nucleolar processing events and methylates 18S rRNA after shuttling of pre-40S with bound Dim1 in the cytosol as shown for the yeast rRNA dimethylase Dim1 (Lafontaine *et al.*, 1994, 1995, 1998; Zemp and Kutay, 2007). The suggested function of Arabidopsis Dim1A as a nucleolar/cytoplasmatic rRNA dimethylase is supported by its position on the phylogenetic tree where it appears to be most closely related to other eukaryotic rRNA dimethylases like ScDim1. Our search detected Dim1 homologues in all completely sequenced plant genomes from algae to flowering plants suggesting that the 18S rRNA of cytoplasmic ribosomes might generally be a target for methylation in plants.

Dim1B was found to possess a mitochondrial transit peptide. Further results strongly support the suggestion that Dim1B is a mitochondrial 18S rRNA adenosine dimethyltransferase. Firstly, we found that recombinant Arabidopsis Dim1B is capable of correctly methylating *E. coli* 16S rRNA at the conserved sites in *ksgA* mutant strains lacking the 16S rRNA methyltransferase activity of KsgA. Secondly, while we observed adenosine modification of mitochondrial 18S rRNA at the conserved site in wild-type Arabidopsis, we found no indication of mitochondrial 18S rRNA methylation in an Arabidopsis mutant line bearing a T-DNA insertion in the *Dim1B* gene, suggesting that the lack of mitochondrial 18S rRNA methylation is caused by the missing Dim1B activity.

The methylation of bacterial 16S rRNA is commonly discussed as contributing to the fidelity of translation. Recent reports, however, suggest a role in ribosome assembly. KsgA interacts with small ribosomal subunits near the binding sites of initiation factor 3 and the 50S subunit (Xu *et al.*, 2008). Unassembled ribosomal subunits accumulated in an *E. coli* mutant lacking KsgA. Interestingly, this defect was exacerbated by growth at low temperature, i.e. the *ksgA* mutant exhibited a cold-sensitive phenotype (Connolly *et al.*, 2008). While the absence of methylation of chloroplast 16S rRNA has no influence on growth of the Arabidopsis *Pfc1* mutant under normal temperature, growth of the *pcf1* mutant at low temperature led to impaired chloroplast development (Tokuhisa *et al.*, 1998). In mouse mitochondria, dramatic, even lethal effects were reported from the loss of adenine dimethylation of 12S rRNA. This defect was shown to impair the stability of the small ribosomal subunit, ribosome assembly and translation (Metodiev *et al.*, 2009). In striking contrast, lack of adenine dimethylation of 18S rRNA in Arabidopsis mitochondria has no obvious effect on growth and development of the investigated T-DNA insertion line, i.e. this conserved rRNA methylation seems not to be essential for Arabidopsis under the conditions of growth in our experiments. This conclusion is also supported by an unaltered phenotype of the *dim1B* mutant under cold stress (data not shown).

Several observations suggest that mitochondrial transcription in Arabidopsis requires auxiliary factors: (i) RpoTmp, the dual-targeted phage-type RNA polymerase, shows virtually no specific *in vitro* transcription initiation regardless of the promoter offered, (ii) RpoTp and RpoTm initiate transcription correctly *in vitro* only with some of the tested promoters; and (iii) correct *in vitro* transcription initiation by the RNA polymerases RpoTp or RpoTm is only observed if the DNA template is in a supercoiled conformation (Kühn *et al.*, 2007). Similar observations have previously been reported for the mitochondrial RNA polymerase Rpo41 from yeast which recognizes mitochondrial promoters on supercoiled, but not linear DNA templates *in vitro* (Matsunaga and Jaehning, 2004). Complementation with sc-mtTFB, however, did enable promoter-specific initiation of transcription on linear templates (Matsunaga and Jaehning, 2004). The results of the *in vitro* experiments performed in this study indicate that Dim1B does not have a function in transcription analogous to sc-mtTFB. Addition of Dim1B to the transcription assays did not lead to correct initiation of transcription by RPOTmp with supercoiled or linear template DNAs whatever Arabidopsis mitochondrial promoter was offered. Moreover, Dim1B assisted RPOTm neither in using additional promoters on supercoiled templates nor in recognizing any promoter on linear templates.

mtTFBs are essential transcription factors in fungal and animal mitochondria (Falkenberg *et al.*, 2007). A similar role of Dim1B as a transcription factor in Arabidopsis mitochondria would not be compatible with our finding that a mutant line lacking functional Dim1B grows and develops normally. Microarray analyses revealed no significant down- or up-regulation of the expression of mitochondrial genes in the *dim1B* mutant in comparison with the wild type (data not shown). Moreover, the positioning of Dim1B in phylogenetic trees supports the conclusion that Dim1B acts as an rRNA dimethyltransferase but not as a mitochondrial transcription factor. The group of predicted mitochondrial rRNA dimethylases, which is only found in flowering plants and includes Arabidopsis Dim1B, does not branch together with mtTFB sequences but appears as a sister group of all eukaryotic Dim1-like proteins.

It is very likely that the evolutionary processes leading to the recruitment of an rRNA dimethylase as auxiliary factor of the mitochondrial transcription machinery in fungi and animals took place after the separation of the common ancestor of these groups from the green algae and plant lineage (Figures 2 and S2; for eukaryotic phylogeny, see (Burger *et al.*, 2003). The position of Arabidopsis Pfc1 and other plant Pfc1 homologues in the phylogram (Figures 2 and S2) is similarly inconclusive with respect to a possible additional role of these proteins as cofactors of phage-type RNA polymerases in plastids. Pfc1 appears to be separated from the Dim1 and mtTFB groups and to be most closely related to chlamydial KsgA homologues. This is in congruence with data from a recent publication (Park *et al.*, 2009) where implications of this positioning are discussed.

No Dim1B homologues have so far been detected in non-flowering plants, raising the question of whether mitochondrial small-subunit rRNAs in algae and in deep branching land plants such as mosses and lycopods lack methylation like the yeast 15S rRNA. In plants without a Dim1B homologue, Dim1A or Pfc1 may be recruited additionally to mitochondria by dual targeting of these proteins. Predictions of the subcellular localization of algal Pfc1-like proteins yield equally high scores for plastidial and mitochondrial localization (Table 1). On the other hand, two Dim1A-like proteins are found in the fully sequenced genome of *P. patens*, with PpDim1A-2 being predicted to have an N-terminal mitochondrial transit peptide (Table 1), suggesting that this protein might replace Dim1B in its function as a mitochondrial rRNA dimethylase.

In conclusion, we found no evidence for a role of Dim1B in mitochondrial transcription in Arabidopsis. Instead, our data provide conclusive evidence of Dim1B functioning as a bona fide rRNA dimethyltransferase methylating the mitochondrial 18S rRNA. The observation of genes orthologous to *Dim1B* in the genome of other angiosperms suggests that adenine dimethylation of mitochondrial 18S rRNA is a common phenomenon among higher plants, the functional importance of which remains to be investigated.

## Experimental procedures

### Plant material and growth condition

*Arabidopsis thaliana* (ecotype Columbia) wild type and T-DNA insertion line 071F10 were grown under short-day conditions (8 h of light/16 h of dark) for 14 days and then transferred to long-day conditions (16 h of light/8 h of dark) in a growth chamber.

### Analysis of an Arabidopsis *dim1B* mutant

The T-DNA insertion line 071F10 was obtained from the GABI-Kat collection. Plants homozygous for the mutation were identified by PCR analysis of a segregating population. Segregation analysis showed the mutant trait to be inherited in a monogenic fashion (3:1 segregation). Sequencing of T-DNA borders identified the insertion site within exon 2 of the genomic *Dim1B* sequence on chromosome V (At5g66360), 947 nucleotides downstream of the ATG initiation start codon in the CDS (see Figure S5). From the selfed line, we selected a homozygous mutant by PCR analysis and named it *dim1B*. The presence of a single T-DNA insertion in *dim1B* was shown by Southern blot analysis of the mutant using the T-DNA probe (data not shown).

### Isolation of RNA from Arabidopsis

Total cellular RNA was extracted from leaves and flowers of Arabidopsis plants using TRIZOL (Invitrogen, http://www.invitrogen.com/) according to the protocol provided by the manufacturer. Total cDNA was synthesized from 1 μg mRNA-enriched Arabidopsis RNA using Poly(A)Purist™ kit (Ambion, http://www.ambion.com) and Omniscript RT kit (QIAGEN, http://www.qiagen.com/) according to the manufacturer’s instructions. Protein-coding sequences were amplified from the Arabidopsis cDNA with *Pfu* DNA polymerase (Promega, http://www.promega.com/). Sanger dideoxy sequencing was performed on an ABI3130xl sequencer (Applied Biosystems, http://www.appliedbiosystems.com/).

### Generation of targeting constructs, transient expression and confocal microscopy

To generate the pDim1B–GFP and pDim1A–GFP constructs driving the expression of fusion proteins Dim1B–GFP and Dim1A–GFP, sequences encoding the 64 N-terminal amino acids of Dim1B and the complete coding sequence of Dim1A were amplified from reverse-transcribed mRNA-enriched Arabidopsis RNA using primer pairs gfp-Dim1B-P/gfp-Dim1B-M (5′-cagctctagaATGATTCTTCGATTGAAAGACCA-3′/5′-cagcgtcgacTGCACAGAAACAATCCATCG-3′) and gfp-Dim1A-P/gfp-Dim1A-4M (5′-cagctctagaATGGCGGGAGGCAAGATC-3′/5′-cagcgtcgacTGAAGTGAATACCAGACTTGTTACA-3′), respectively. PCR products were *Xba*I/*Sal*I-digested and inserted into the *Spe*I/*Sal*I-cleaved vector pOL–GFP S65C (Peeters *et al.*, 2000). Control constructs encoding mitochondrial Cox4–GFP and plastidial RecA–GFP were kindly provided by I. Small (Peeters *et al.*, 2000).

GFP-constructs were used to transform isolated Arabidopsis protoplasts as described in Yin *et al.* (2009). Protoplasts were examined 2 days after transformation by confocal laser scanning microscopy with a Leica TCS SP2 using 488 nm excitation and two-channel measurement of emission from 510 to 580 nm (green/GFP) and >590 nm (red/chlorophyll).

### Sequence data and multiple alignments

Genomic sequences, EST sequences and amino acid sequences were retrieved from the National Center for Biotechnology Information (http://www.ncbi.nlm.nih.gov/BLAST/) employing the BLASTP and TBLASTN algorithms and from the Joint Genome Institute (http://genome.jgi-psf.org/). *E. coli* KsgA, human Dim1, AtPfc1 and h-TFB1M sequences were used as query sequences. For translation and alignment, sequences were subsequently imported into geneious v4.5 (Drummond *et al.*, 2008). A multiple protein sequence alignment was generated using clustalw (Thompson *et al.*, 1994) implemented in the geneious package using GONNET cost matrix with gap open cost set to 10 and gap extend cost set to 0.1. The alignment was refined manually with respect to the known secondary structural homology and poorly aligned regions were excluded from following analyses.

For a phylogenetic reconstruction based on Bayesian statistics, the MrBayes program version 3.1 (Ronquist and Huelsenbeck, 2003) was used. In addition, maximum likelihood (phyML; Guindon and Gascuel, 2003) and neighbour joining analysis (Saitou and Nei, 1987) implemented in the geneious v4.5 package were conducted.

### Plasmids for the expression of recombinant proteins

Sequences encoding amino acids 18–353 of Dim1A (AGI At2g47420) and amino acids 26–380 of Dim1B (AGI At5g66360) were amplified from reverse-transcribed mRNA-enriched Arabidopsis RNA using oligonucleotide primer pairs Dim1A-PROP/Dim1A-PROM (cagcgtcgacTCGAACCATTACCAAGGAGGAATAT/cagcgcggccgcACACCACAAAACGATTATGTGAAGTG) and Dim1B-PROP/Dim1B-PROM (cagcgtcgacCGAGATTCTCACTCGCAGGC/cagcgcggccgcTTATTCGTGTAGATCCATTTGTAATGATG), respectively. PCR products were ligated into pPROTet.E121 (Clontech, http://www.clontech.com/) to generate plasmids pPRO-Dim1A and pPRO-Dim1B.

### Poisoned primer extension

Primer extension reactions were carried out according to standard protocols (Sambrook *et al.*, 1989) using 10 μg of total Arabidopsis RNA isolated from wild-type plants and the homozygous dim1B insertion line each. The DNA oligonucleotide primer PPE*Atmt18S* (5′-GAAGGATTCAATCCAGCCACA-3′) was end-labelled with [γ-^32^P]ATP and T4-polynucleotide kinase (Fermentas, http://www.fermentas.com). Primer extensions were performed using Superscript-III MMLV reverse transcriptase (Invitrogen) at 50°C, with nucleotides ddATP, dCTP, dGTP, dTTP at 0.02 mm and with 2 pmol end-labelled primer. The end-labelled oligonucleotide 5′-TAAGGAGGTGATCCAACCGCA-3′ was used for reverse transcription for the analysis of the AA dinucleotide at the bacterial rRNA nucleotides A1518 and A1519, the positions equivalent to the plant mitochondrial rRNA nucleotides A1914 and A1915. RNA sequencing reactions were performed as described previously (Lowe and Eddy, 1999) using total RNA of strains JM101*KsgA19* and BL21(DE2)*KsgA_0*, respectively. Resulting products were analysed on a 10% sequencing gel. Visualization was done with a PhosphoImaging system and the complementary software (Bio-Rad, http://www.bio-rad.com/).

### Complementation of bacterial KsgA knock-out mutants

For the bacterial complementation tests, *E. coli* strains JM101*KsgA19* and BL21(DE3)*KsgA_0* lacking the *KsgA* gene and JM101 were kindly provided by Dr A. Vila-Sanjurjo. The *Dim1B* gene lacking the N-terminal targeting signal was ligated into pBluescriptII KS (pBS_Dim1B) and into the *Bam*HI and *Xho*I sites of pET28a (pET28_ Dim1B) and pET29a (pET29_ Dim1B) (Novagen, http://www.novagen.com), respectively. Bacterial strains Strain BL21(DE3)pLys (Stratagene, http://www.stratagene.com/), as well as JM101, were used as positive controls. Strain JM101*KsgA19* was transformed with pBS_ Dim1B. Strain BL21(DE2)*KsgA_0* was transformed with pET28_ Dim1B and pET29_ Dim1B, respectively. Each bacterial strain was grown up to A_600nm_∼0.6 and total RNA was isolated using a previously described method (Khodursky *et al.*, 2003).

### Protein expression and purification

Recombinant Dim1A and Dim1B were overexpressed in *Escherichia coli* BL21 Codon Plus RIL (Stratagene), purified over Ni^2+^–NTA–agarose (QIAGEN) and stored in 20 mm Tris/HCl, pH 7.8, 50 mm NaCl, 0.5 mm EDTA, 1 mm DTT, 50% (v/v) glycerol at −20°C for 2–6 weeks. Expression and purification of recombinant RPOTm and RPOTmp was performed as described (Kühn *et al.*, 2007).

### *In vitro* transcription assay

To generate DNA templates for *in vitro* transcription assays, mitochondrial DNA fragments were ligated into pKL23 (Liere and Maliga, 1999) upstream of terminator sequences; all plasmids used in this study have been described earlier (Kühn *et al.*, 2007). Linearized templates were generated by *Xho*I or *Eco*RI digests and purified via QIAquick spin columns (QIAGEN).

*In vitro* transcription assays were carried out for 45 min at 30°C essentially as described in (Kühn *et al.*, 2007). Reactions contained 6.7 mm Tris/HCl (pH 7.9), 6.7 mm KCl, 6.7 mm MgCl_2_, 0.67 mm DTT, 0.067% (w/v) BSA, 267 μm each ATP, CTP and GTP, 13 μm unlabelled UTP and 10 μCi of [γ-^32^P]–UTP (3000 Ci/mmol), 24 U RNase inhibitor and 200 ng of template DNA in a final volume of 15 μl. Reactions were started by adding 400 fmol of recombinant RPOTm or RPOTmp and 400 fmol of recombinant Dim1A or Dim1B where indicated and stopped with 115 μl RNA extraction buffer [6 m urea, 360 mm NaCl, 20 mm EDTA, 10 mm Tris/HCl pH 8.0, 1% (w/v) SDS] and 20 μl 2.25 m sodium acetate (pH 5.2). Nucleic acids were extracted with phenol/chloroform/isoamyl alcohol (25:24:1) and ethanol precipitated. Transcripts were then dissolved in formamide buffer [95% (v/v) formamide; 0,02% (w/v) bromphenol blue; 0,02% (w/v) xylene cyanol] and resolved using a Protean II xi electrophoresis unit (Bio-Rad) on 7 m urea, 5% polyacrylamide gels using 0.6× TBE as electrophoresis buffer. A radiolabeled RNA length standard was generated using the RNA Century Marker Template Plus (Ambion) and MAXIscript kit (Ambion) according to the manufacturer’s instructions and separated alongside RNA samples. Following electrophoresis, gels were dried and subjected to autoradiography employing a phosphoimager (Molecular Imager FX; Bio-Rad).

## SUPPORTING INFORMATION

**Figure S1.** Alignment of bacterial and eukaryotic rRNA methyltransferases. Eight sequence motifs conserved in rRNA methyltransferases are labelled below the alignment; secondary structure elements are shown above, with α-helices indicated in grey and β-sheets in black [taken from O’Farrell *et al.*, (2004) and Shutt and Gray (2006a)]. Shading of single residues based on similarity scores in each column is as follows: 100% black; >80% dark grey; >60% light grey; <60% white. Genomic sequences, EST sequences and amino acid sequences were retrieved from the National Center for Biotechnology Information (http://www.ncbi.nlm.nih.gov/BLAST/) employing the BLASTP and TBLASTN algorithms and from the Joint Genome Institute (http://genome.jgi-psf.org/). *E. coli* KsgA, human Dim1, AtPFC1 and h-TFB1M sequences were used as query sequences. For translation and alignment, sequences were subsequently imported into geneious v4.5 (Drummond *et al.*, 2008). A multiple protein sequence alignment was generated using clustalw (Thompson *et al.*, 1994) implemented in the geneious package using GONNET cost matrix with gap open cost set to 10 and gap extend cost set to 0.1. The alignment was refined manually with respect to the known secondary structural homology and poorly aligned regions were excluded from following analyses.

**Figure S2.** Phylogeny of mitochondrial transcription factors and small-subunit rRNA dimethylases. The phylogeny was reconstructed by Bayesian estimation from conserved amino acid sequence sections indicated in the amino acid sequence alignment (see Supporting Information Figure S1). The aligned sequence data comprised 212 amino acids in length. Of these character states, 210 were parsimony informative. For a reconstruction based on Bayesian statistics, the MrBayes program version 3.1 (Ronquist and Huelsenbeck, 2003) was used. The Bayesian inference method employed the wag amino acid replacement model and a gamma distribution to represent among-site rate heterogeneity (wag +γ). A discrete gamma distribution with four categories was assumed to approximate the continuous function. The Metropolis-coupled Markov chain Monte Carlo analysis (MCMC) was performed with 3.5 million generations and three independent chains in two runs. The Markov chain was sampled every 100 generations. Final average standard deviation of split frequencies was 0.015. Additionally convergence was judged by plots of maximum likelihood (ML) scores and by using the run statistics. The first 25% of all trees were discarded; the remaining trees were used to construct a consensus tree and to calculate the posterior branch support values. Essentially the same tree topology was obtained employing maximum likelihood (phyML (Guindon and Gascuel 2003)) and neighbour-joining analysis (Saitou and Nei 1987) implemented in the geneious v4.5 package. Clusters are coloured as follows: Plant Dim1A (white branch), Dim1B (red branch) and other eukaryotic Dims (blue branch) in pale blue, archaeal proteins in orange, cyanobacterial proteins pastel green, other bacterial proteins rose, chlamydial and plant Pfc (dark green branch) proteins in lime-green, chromatibacterial proteins in yellow, mitochondrial transcription factors (purple branches) and alphaproteobacterial sequences in magenta. Prefixes of designations of plant Dim and Pfc-like proteins refer to *Arabidopsis thaliana* (At), *Populus trichocarpa* (Pt), *Glycine max*. (Gm), *Medicago truncatula* (Mt), *Solanum lycopersicum* (Sl), *Nicotiana benthamiana* (Nben), *Vitis vinifera* (Vv), *Sorghum bicolor* (Sb), *Oryza sativa* (Os), *Zea mays* (Zm), *Triticum aestivum* (Ta), *Picea sitchensis* (Ps), *Pinus taeda* (Ptae), *Marchantia polymorpha* (Mp), *Physcomitrella patens* (Pp), *Selaginella moellendorffii* (Sm), *Chlamydomonas reinhardtii* (Cr), *Volvox carteri* f. nagariensis (Vc), *Chlorella vulgaris* C-169 (Cv), *Ostreococcus tauri* (Ot), *Ostreococcus lucimarinus* (Ol), *Thellungiella halophila* (Th), *Gossypium hirsutum* (Gh), *Welwitschia mirabilis* (Wm), *Picea glauca* (Pg), *Micromonas pusilla* (Mpul). *Cyanidioschyzon merolae* is designated by Cm. Fungal and animal protein prefixes refer to *Kluyveromyces lactis* (Kl), *Ashbya gossypii* (Ago), *Saccharomyces cerevisiae* (Sc), *Saccharomyces douglasii* (Sd), *Saccharomyces kluyveri* (Sk), *Candida albicans* (Ca), *Candida glabrata* (Cg), *Pichia stipitis* (Psi), *Debaryomyces hansenii* (Dh), *Schizosaccharomyces pombe* (sp), *Ustilago maydis* (Um), *Homo sapiens* (Hs), *Mus musculus* (Mm), *Rattus norvegicus* (Rn), *Gallus gallus* (Gg), *Xenopus laevis* (Xl), *Danio rerio* (Dr), *Strongylocentrotus purpuratus* (St), *Drosophila melanogaster* (Dm), *Anopheles gambiae* (Ag). EcKsga and PaeKsgA are 16 S rRNA dimethylases from *Escherichia coli* and *Pseudomonas aeruginosa* respectively. Numbers at branching points are posterior branch support values. Branch lengths correspond to the number of inferred amino acid changes per position, as indicated by the scale bar.

**Figure S3.** Expression of recombinant Dim1A and Dim1B and analysis of their DNA binding capacity by electrophoretic mobility shift assays (EMSAs). (a) DIM1B and DIM1A fused to N-terminal hexahistidine tags were expressed in *E. coli*, purified over Ni2 + -NTA agarose and analysed by SDS-PAGE followed by Coomassie-blue staining of the gel (left panel). Samples were run alongside a molecular weight marker; sizes are indicated in kDa (marker lane not displayed). Proteins of the expected size were found to be enriched and their identity was confirmed by western blotting and immunolabelling with an anti-polyhistidine antibody (right). (b) EMSAs testing DNA binding by DIM1A and DIM1B. Binding reactions were set up essentially as described by (24) with 50 or 100 ng of recombinant DIM1B (left panel) or DIM1A (right panel) and with 5’ end-labelled mitochondrial DNA fragments containing the P*atp*9-295 or the P*atp*-239 promoter (see below) as denoted by the above the autoradiographs. The addition of increasing amounts (5 and 10 ng) of the non-specific competitor poly(dI-dC) to binding reactions is indicated below. Binding reactions were subjected to non-denaturing PAGE. Signals corresponding to DNA–protein complexes (C) and unbound DNA (F) are marked. (c) Linear diagram of the upstream region of the mitochondrial gene *atp9* displaying two typical mitochondrial promoters, P*atp*9-295 and P*atp*-239. Grey lines mark the position of two mitochondrial fragments used as probes in EMSAs; core sequences of the comprised promoters are shown. Positions of promoters and transcriptional starts (bent arrows) and processing sites (triangles) are given as negative distances (in base pairs) from the translational start equalling position +1, which is highlighted by a filled circle.

**Figure S4.** Run-off transcription from the mitochondrial *atp6-1*, *atp8* and *rrn18* upstream regions by RPOTm and RPOTmp. (a) RPOTm and RPOTmp were assayed for promoter-specific transcription from plasmids pKL23-*atp6-1*, pKL23-*atp8* and pKL23-*rrn18* provided as supercoiled (lanes labelled ccc) or linearized DNA templates (lin). *In vitro* transcription reactions were run in the presence or absence of Dim1B (2) or Dim1A (1) as indicated. Labelled RNA products were separated in a denaturing polyacrylamide gel alongside an RNA size marker; sizes are given in nucleotides (indicated on the left). Major discrete RNA products obtained with supercoiled templates are indicated by arrows and, according to our previous *in vitro* transcription analyses (Kühn *et al.*, 2007), attributed to transcription initiation at P*atp6-1*-200 followed by termination at terminator sequences *hisa* (signals labelled P-200-*hisa*) or *thra* (signal P-200-*thra*), initiation at Patp8-228/226 and termination at *hisa* or *thra* and initiation at P*rrn18*-156 and termination at *hisa* or *thra*. Extended expositions of gels did not reveal additional bands. (b) DNA templates were constructed by inserting *Arabidopsis* mitochondrial promoter regions (grey bars) into pKL23 (Liere and Maliga, 1999) upstream of the two bacterial ρ-independent terminator sequences *hisa* (Barnes and Tuley, 1983) and *thra* (Gardner, 1982). When providing a supercoiled pKL23 derivative as template, transcription initiated at the introduced promoters is terminated at *hisa* and/or *thra*, thereby generating RNA products of distinct lengths. Transcripts expected from initiation at mitochondrial promoters P*atp6-1*-156, P*atp6-1*-200, P*atp8*-157, P*atp8*-228/226, P*rrn18*-69 or P*rrn18*-156 (bent arrows) and termination at *hisa* or *thra* (triangles) or at the restriction sites *Eco*RI (E) used for linearization of pKL23-*atp6-1* or *Xho*I (X) used for pKL23-*atp8* and pKL23-*rrn18* linearization are indicated by horizontal black arrows labelled with the respective RNA lengths. Minor signals indicated by grey arrows labelled with asterisks may be due to differently migrating major products.

**Figure S5.** Schematic representation of T-DNA insertion in *dim1B* plants. (a) Schematic representation of T-DNA insertion 071F10. Location of primers is indicated by arrows. (b) PCR analysis of a segregating *Dim1B* population. PCR was performed with gene-specific primers gsp-p (GGCCTTATTGTTGTTGCTAGGATTG) and gsp-m (CTTCTCTTCGAACCCATTTGTTCTC) to detect the wild-type allele and with primers gsp-p and LB (T-DNA left border, CCCATTTGGACGTGAATGTAGACAC) to detect the T-DNA insertion.

**Table S1.** Physicochemical properties and targeting prediction results for Arabidopsis Dim1A (At2g474420) and Dim1B (At5g666360).

**Table S2.** Promoter sequences analysed in an *in vitro* transcription assay.

References S1

As a service to our authors and readers, this journal provides supporting information supplied by the authors. Such materials are peer-reviewed and may be re-organized for online delivery, but are not copy-edited or typeset. Technical support issues arising from supporting information (other than missing files) should be addressed to the authors.

## References

[b1] Asin-Cayuela J, Gustafsson CM (2007). Mitochondrial transcription and its regulation in mammalian cells. Trends Biochem. Sci..

[b2] Binder S, Brennicke A (2003). Gene expression in plant mitochondria: transcriptional and post-transcriptional control. Philos. Trans. R. Soc. Lond. B Biol. Sci..

[b3] Bogenhagen DF (1996). Interaction of mtTFB and mtRNA polymerase at core promoters for transcription of *Xenopus laevis* mtDNA. J. Biol. Chem..

[b4] Bogenhagen DF, Insdorf NF (1988). Purification of *Xenopus laevis* mitochondrial RNA polymerase and identification of a dissociable factor required for specific transcription. Mol. Cell. Biol..

[b5] Burger G, Gray MW, Lang BF (2003). Mitochondrial genomes: anything goes. Trends Genet..

[b6] Cermakian N, Ikeda TM, Cedergren R, Gray MW (1996). Sequences homologous to yeast mitochondrial and bacteriophage T3 and T7 RNA polymerases are widespread throughout the eukaryotic lineage. Nucleic Acids Res..

[b7] Connolly K, Rife JP, Culver G (2008). Mechanistic insight into the ribosome biogenesis functions of the ancient protein KsgA. Mol. Microbiol..

[b8] Corpet F (1988). Multiple sequence alignment with hierarchical clustering. Nucleic Acids Res..

[b9] Cotney J, Shadel GS (2006). Evidence for an early gene duplication event in the evolution of the mitochondrial transcription factor b family and maintenance of rrna methyltransferase activity in human mtTFB1 and mtTFB2. J. Mol. Evol..

[b10] Cotney J, Wang Z, Shadel GS (2007). Relative abundance of the human mitochondrial transcription system and distinct roles for h-mtTFB1 and h-mtTFB2 in mitochondrial biogenesis and gene expression. Nucleic Acids Res..

[b11] Drummond A, Ashton B, Cheung M, Heled J, Kearse M, Moir R, Stones-Havas S, Thierer T, Wilson A (2008). Geneious v4.0.

[b12] Falkenberg M, Gaspari M, Rantanen A, Trifunovic A, Larsson N, Gustafsson C (2002). Mitochondrial transcription factors B1 and B2 activate transcription of human mtDNA. Nat. Genet..

[b13] Falkenberg M, Larsson NG, Gustafsson CM (2007). DNA replication and transcription in mammalian mitochondria. Annu. Rev. Biochem..

[b14] Fisher RP, Clayton DA (1988). Purification and characterization of human mitochondrial transcription factor 1. Mol. Cell. Biol..

[b15] Gaspari M, Falkenberg M, Larsson NG, Gustafsson CM (2004). The mitochondrial RNA polymerase contributes critically to promoter specificity in mammalian cells. EMBO J..

[b16] Gray MW, Burger G, Lang BF (1999). Mitochondrial evolution. Science.

[b17] Greenleaf AL, Kelly JL, Lehman IR (1986). Yeast RPO41 gene product is required for transcription and maintenance of the mitochondrial genome. Proc. Natl Acad. Sci. USA.

[b18] Guindon S, Gascuel O (2003). A simple, fast and accurate algorithm to estimate large phylogenies by maximum likelihood. Syst. Biol..

[b19] Hagenbüchle O, Santer M, Steitz JA, Mans RJ (1978). Conservation of the primary structure at the 3′ end of 18S rRNA from eucaryotic cells. Cell.

[b20] Hedtke B, Börner T, Weihe A (1997). Mitochondrial and chloroplast phage-type RNA polymerases in Arabidopsis. Science.

[b21] Hedtke B, Börner T, Weihe A (2000). One RNA polymerase serving two genomes. EMBO Rep..

[b22] Herrmann JM (2003). Converting bacteria to organelles: evolution of mitochondrial protein sorting. Trends Microbiol..

[b23] Jang SH, Jaehning JA (1991). The yeast mitochondrial RNA polymerase specificity factor, MTF1, is similar to bacterial sigma factors. J. Biol. Chem..

[b24] Kanki T, Ohgaki K, Gaspari M, Gustafsson CM, Fukuoh A, Sasaki N, Hamasaki N, Kang D (2004). Architectural role of mitochondrial transcription factor A in maintenance of human mitochondrial DNA. Mol. Cell. Biol..

[b25] Khodursky AB, Bernstein JA, Peter BJ, Rhodius V, Wendisch VF, Zimmer DP (2003). Escherichia coli spotted double-strand DNA microarrays: RNA extraction, labeling, hybridization, quality control and data management. Methods Mol. Biol..

[b26] Kühn K, Bohne A-V, Liere K, Weihe A, Börner T (2007). *Arabidopsis* single-polypeptide RNA polymerases: accurate in vitro transcription of organellar genes. Plant Cell.

[b27] Lafontaine D, Delcour J, Glasser AL, Desgres J, Vandenhaute J (1994). The *DIM1* gene responsible for the conserved m6(2)Am6(2)A dimethylation in the 3′-terminal loop of 18 S rRNA is essential in yeast. J. Mol. Biol..

[b28] Lafontaine D, Vandenhaute J, Tollervey D (1995). The 18S rRNA dimethylase Dim1p is required for pre-ribosomal RNA processing in yeast. Genes Dev..

[b29] Lafontaine DL, Preiss T, Tollervey D (1998). Yeast 18S rRNA dimethylase Dim1p: a quality control mechanism in ribosome synthesis?. Mol. Cell. Biol..

[b30] Liere K, Maliga P (1999). In vitro characterization of the tobacco rpoB promoter reveals a core sequence motif conserved between phage-type plastid and plant mitochondrial promoters. EMBO J..

[b31] Lowe TM, Eddy SR (1999). A computational screen for methylation guide snoRNAs in yeast. Science.

[b32] Margulis L (1970). Origin of Eukaryotic Cells.

[b33] Margulis L (1981). Symbiosis in Cell Evolution.

[b34] Masters BS, Stohl LL, Clayton DA (1987). Yeast mitochondrial RNA polymerase is homologous to those encoded by bacteriophages T3 and T7. Cell.

[b35] Matsunaga M, Jaehning JA (2004). Intrinsic promoter recognition by a ‘core’ RNA polymerase. J. Biol. Chem..

[b36] Matsushima Y, Garesse R, Kaguni LS (2004). Drosophila mitochondrial transcription factor B2 regulates mitochondrial DNA copy number and transcription in schneider cells. J. Biol. Chem..

[b37] Matsushima Y, Adan C, Garesse R, Kaguni LS (2005). Drosophila mitochondrial transcription factor B1 modulates mitochondrial translation but not transcription or DNA copy number in Schneider cells. J. Biol. Chem..

[b38] McCulloch V, Shadel GS (2003). Human mitochondrial transcription factor B1 interacts with the C-terminal activation region of h-mtTFA and stimulates transcription independently of its RNA methyltransferase activity. Mol. Cell. Biol..

[b39] McCulloch V, Seidel-Rogol BL, Shadel GS (2002). A human mitochondrial transcription factor is related to RNA adenine methyltransferases and binds *S*-adenosylmethionine. Mol. Cell. Biol..

[b40] Metodiev MD, Lesko N, Park CB, Camara Y, Shi Y, Wibom R, Hultenby K, Gustafsson CM, Larsson NG (2009). Methylation of 12S rRNA is necessary for in vivo stability of the small subunit of the mammalian mitochondrial ribosome. Cell Metab..

[b41] Newton KJ, Winberg B, Yamato K, Lupold S, Stern DB (1995). Evidence for a novel mitochondrial promoter preceding the *cox2* gene of perennial teosintes. EMBO J..

[b42] Parisi MA, Clayton DA (1991). Similarity of human mitochondrial transcription factor 1 to high mobility group proteins. Science.

[b43] Parisi MA, Xu B, Clayton DA (1993). A human mitochondrial transcriptional activator can functionally replace a yeast mitochondrial HMG-box protein both *in vivo* and *in vitro*. Mol. Cell. Biol..

[b44] Park AK, Kim H, Jin HJ (2009). Comprehensive phylogenetic analysis of evolutionarily conserved rRNA adenine dimethyltransferase suggests diverse bacterial contributions to the nucleus-encoded plastid proteome. Mol. Phylogenet. Evol..

[b45] Peeters N, Small I (2001). Dual targeting to mitochondria and chloroplasts. Biochim. Biophys. Acta.

[b46] Peeters N, Chapron A, Giritch A, Grandjean O, Lancelin D, Lhomme T, Vivrel A, Small I (2000). Duplication and quadruplication of *Arabidopsis thaliana* cysteinyl- and asparaginyl-tRNA synthetase genes of organellar origin. J. Mol. Evol..

[b47] Riemen G, Michaelis G (1993). A point mutation in the core subunit gene of yeast mitochondrial RNA polymerase is suppressed by a high level of specificity factor MTF1. Mol. Gen. Genet..

[b48] Rodriguez-Ezpeleta N, Brinkmann H, Burey SC, Roure B, Burger G, Loffelhardt W, Bohnert HJ, Philippe H, Lang BF (2005). Monophyly of primary photosynthetic eukaryotes: green plants, red algae and glaucophytes. Curr. Biol..

[b49] Ronquist F, Huelsenbeck JP (2003). MrBayes 3: Bayesian phylogenetic inference under mixed models. Bioinformatics.

[b50] Saitou N, Nei M (1987). The neighbor-joining method: a new method for reconstructing phylogenetic trees. Mol. Biol. Evol..

[b51] Sambrook J, Fritsch EF, Maniatis T (1989). Molecular Cloning. A Laboratory Manual.

[b52] Schinkel AH, Groot Koerkamp MJ, Stuiver MH, Van der Horst GT, Tabak HF (1987a). Effect of point mutations on in vitro transcription from the promoter for the large ribosomal RNA gene of yeast mitochondria. Nucleic Acids Res..

[b53] Schinkel AH, Koerkamp MJ, Touw EP, Tabak HF (1987b). Specificity factor of yeast mitochondrial RNA polymerase. Purification and interaction with core RNA polymerase. J. Biol. Chem..

[b54] Schnare MN, Gray MW (1982). 3′-Terminal sequence of wheat mitochondrial 18S ribosomal RNA: further evidence of a eubacterial evolutionary origin. Nucleic Acids Res..

[b55] Schubot FD, Chen CJ, Rose JP, Dailey TA, Dailey HA, Wang BC (2001). Crystal structure of the transcription factor sc-mtTFB offers insights into mitochondrial transcription. Protein Sci..

[b56] Seidel-Rogol BL, McCulloch V, Shadel GS (2003). Human mitochondrial transcription factor B1 methylates ribosomal RNA at a conserved stem-loop. Nat. Genet..

[b57] Shutt TE, Gray MW (2006a). Bacteriophage origins of mitochondrial replication and transcription proteins. Trends Genet..

[b58] Shutt TE, Gray MW (2006b). Homologs of mitochondrial transcription factor B, sparsely distributed within the eukaryotic radiation, are likely derived from the dimethyladenosine methyltransferase of the mitochondrial endosymbiont. Mol. Biol. Evol..

[b59] Sigmund CD, Ettayebi M, Borden A, Morgan EA (1988). Antibiotic resistance mutations in ribosomal RNA genes of *Escherichia coli*. Methods Enzymol..

[b60] Thompson JD, Higgins DG, Gibson TJ (1994). CLUSTAL W: improving the sensitivity of progressive multiple sequence alignment through sequence weighting, position-specific gap penalties and weight matrix choice. Nucleic Acids Res..

[b61] Tiranti V, Savoia A, Forti F, D’Apolito MF, Centra M, Rocchi M, Zeviani M (1997). Identification of the gene encoding the human mitochondrial RNA polymerase (h-mtRPOL) by cyberscreening of the Expressed Sequence Tags database. Hum. Mol. Genet..

[b62] Tokuhisa JG, Vijayan P, Feldmann KA, Browse JA (1998). Chloroplast development at low temperatures requires a homolog of *DIM1*, a yeast gene encoding the 18S rRNA dimethylase. Plant Cell.

[b63] Weihe A, Hedtke B, Börner T (1997). Cloning and characterization of a cDNA encoding a bacteriophage-type RNA polymerase from the higher plant *Chenopodium album*. Nucleic Acids Res..

[b64] Winkley CS, Keller MJ, Jaehning JA (1985). A multicomponent mitochondrial RNA polymerase from *Saccharomyces cerevisiae*. J. Biol. Chem..

[b65] Xu Z, O’Farrell HC, Rife JP, Culver GM (2008). A conserved rRNA methyltransferase regulates ribosome biogenesis. Nat. Struct. Mol. Biol..

[b66] Yang D, Oyaizu Y, Oyaizu H, Olsen GJ, Woese CR (1985). Mitochondrial origins. Proc. Natl Acad. Sci. USA.

[b67] Yin C, Richter U, Borner T, Weihe A (2009). Evolution of phage-type RNA polymerases in higher plants: characterization of the single phage-type RNA polymerase gene from *Selaginella moellendorffii*. J. Mol. Evol..

[b68] Young DA, Lonsdale DM (1997). Evidence that plant mitochondrial transcription requires promoter-specific factors. Maize Genet. Coop. Newsletter.

[b69] Zemp I, Kutay U (2007). Nuclear export and cytoplasmic maturation of ribosomal subunits. FEBS Lett..

