# Supplementary figures and images for: A mitochondrial rRNA dimethyladenosine methyltransferase in Arabidopsis
Source: Plant J. 2010 Feb;61(4):558–69. doi: 10.1111/j.1365-313X.2009.04079.x (PMC2860759; doi:10.1111/j.1365-313X.2009.04079.x)

A

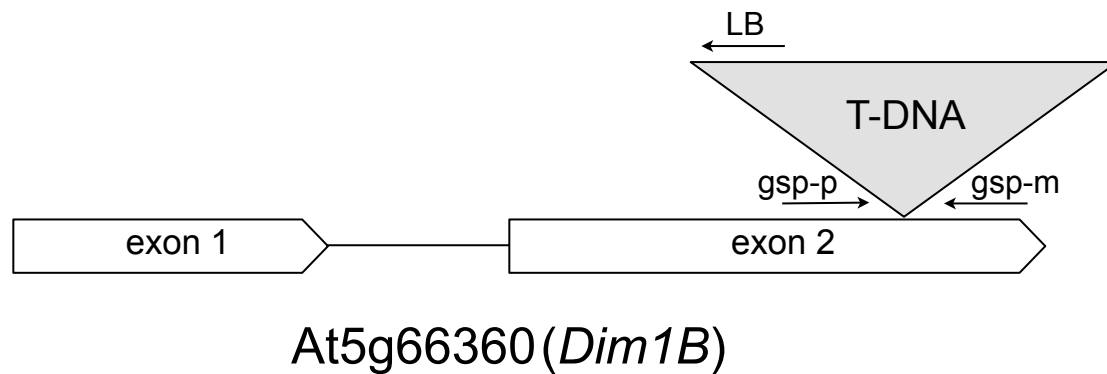

B

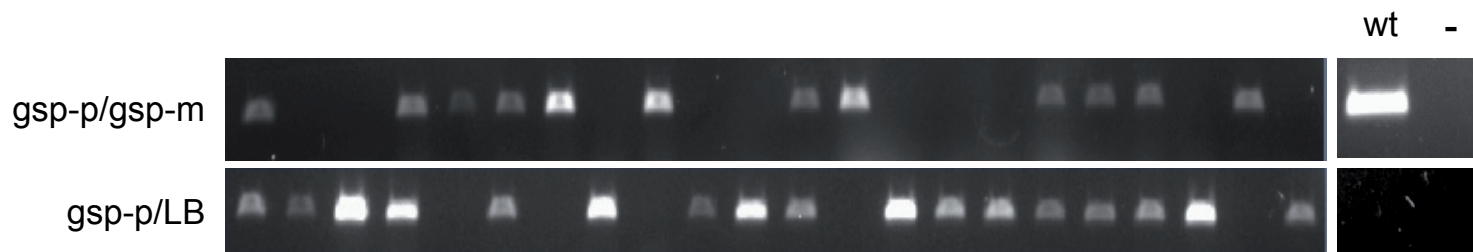

|                     |    |
|---------------------|----|
| wildtype            | 5  |
| heterozygote mutant | 9  |
| homozygote mutant   | 10 |

Supplement: Supplementary file 1 [file tpj0061-0558-SD1.pdf]

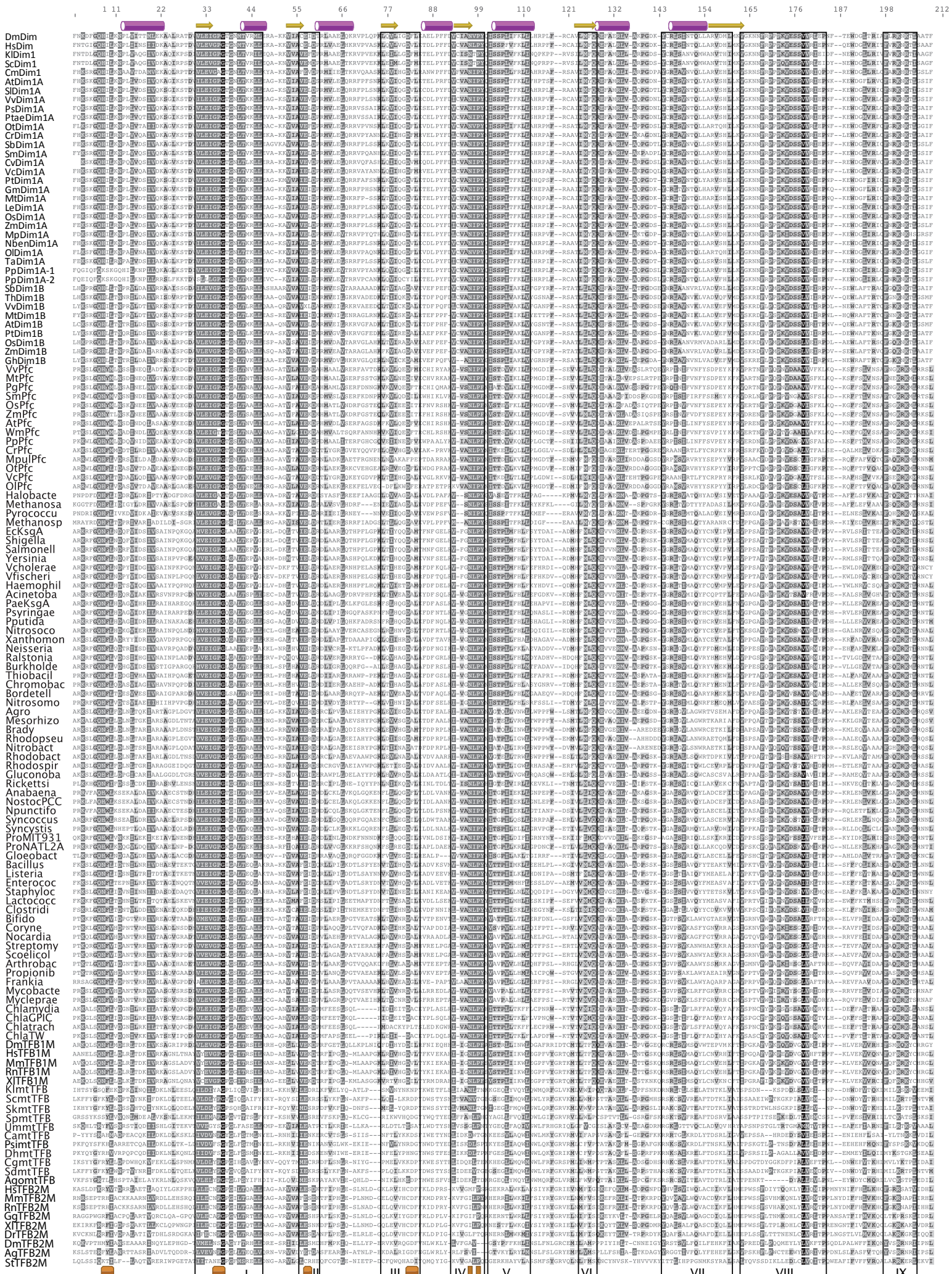

Supplement: Supplementary file 2 [file tpj0061-0558-SD2.pdf]

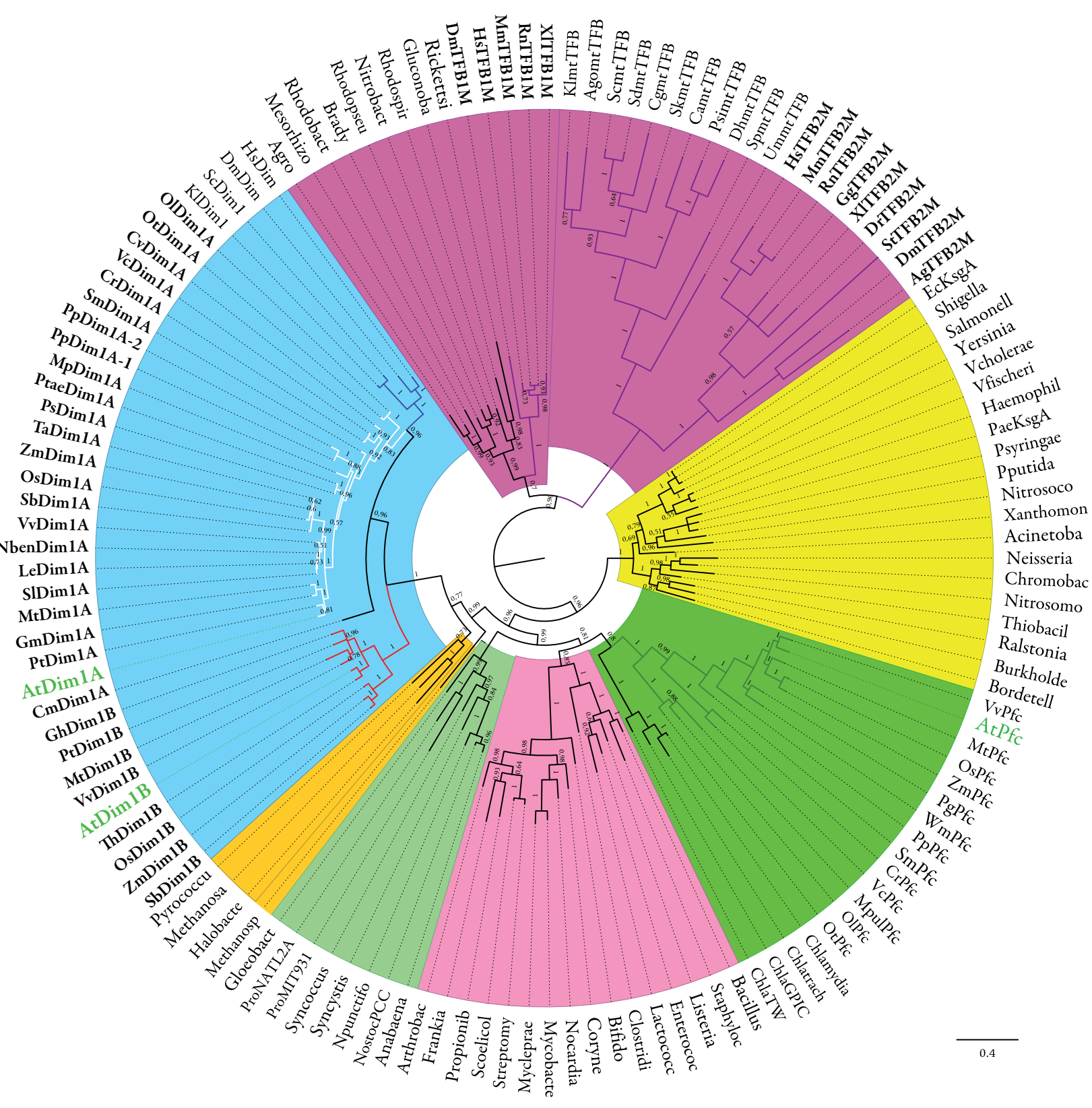

Supplement: Supplementary file 3 [file tpj0061-0558-SD3.pdf]

**A**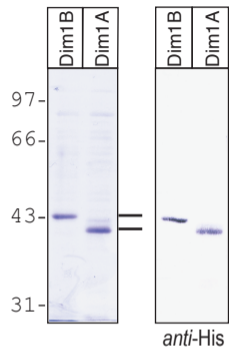**B**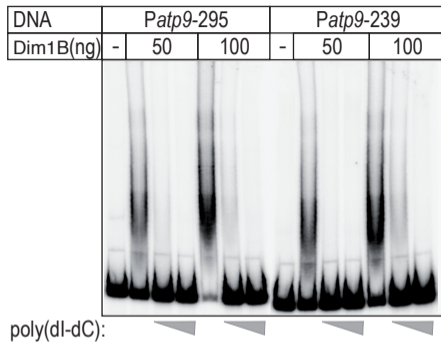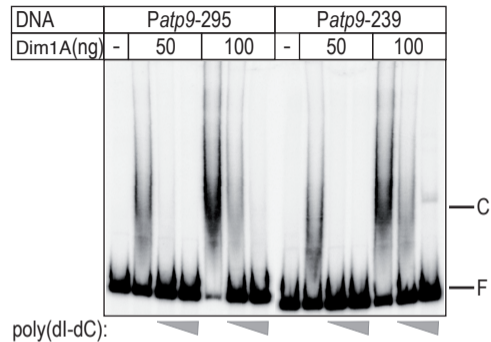**C**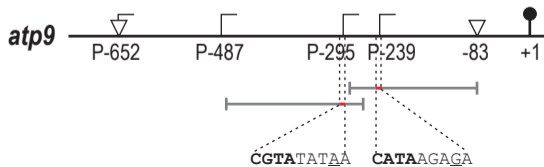

Supplement: Supplementary file 4 [file tpj0061-0558-SD4.pdf]

**A**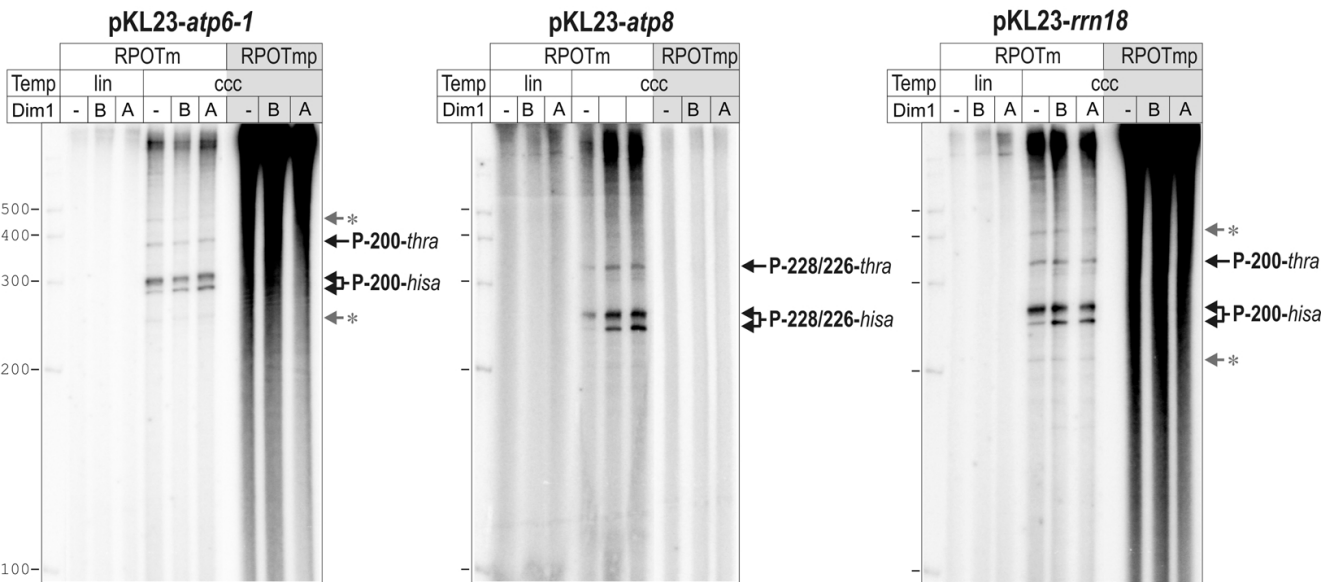**B**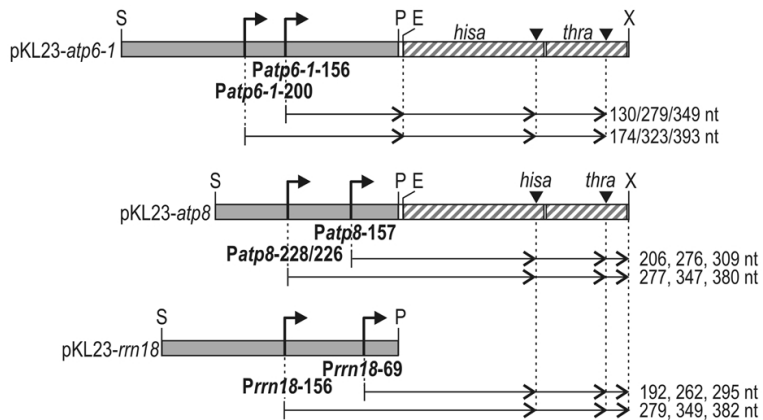

Supplement: Supplementary file 5 [file tpj0061-0558-SD5.pdf]
