# Supplementary material for: A mitochondrial rRNA dimethyladenosine methyltransferase in Arabidopsis
Source: Plant J. 2010 Feb;61(4):558–69. doi: 10.1111/j.1365-313X.2009.04079.x (PMC2860759; doi:10.1111/j.1365-313X.2009.04079.x)
Supplement: Supplementary file 6 [file tpj0061-0558-SD6.doc]

**Supplementary Table S1. Targeting prediction results for Arabidopsis Dim1A (At2g474420) and Dim1B (At5g666360).** Physicochemical properties were derived from Dim1A and Dim1B using ProtParam (<http://expasy.cbr.nrc.ca/tools/protparam.html>). (a): <http://www.cbs.dtu.dk/services/TargetP/>; (b): <http://www.mips.biochem.mpg.de/cgi-bin/proj/medgen/mitofilter>; (c): <http://urgi.versailles.inra.fr/predotar/predotar.html>; (d): <http://psort.ims.u-tokyo.ac.jp/form.html>; (e): <http://hc.ims.u-tokyo.ac.jp/iPSORT/>

| ***Locus tag*** | At2g474420 | At5g666360 |
| --- | --- | --- |
|  |  |  |
| ***Subcellular localization*** |  |  |
| TargetP (a) | not mitochondrial, not plastidial | mitochondrial |
| Mitoprot (b) | non- mitochondrial | mitochondrial |
| Predotar (c) | not mitochondrial, not plastidial | possibly mitochondrial |
| PsortII (d) | nuclear | mitochondrial |
| iPsort (e) | not mitochondrial, not plastidial | mitochondrial |
|  |  |  |
| ***N-terminal transit peptide*** |  |  |
| TargetP | - | 26 aa |
| Mitoprot | - | 27 aa |
| PsortII | - | 27 aa |
|  |  |  |
| ***Mature proteine*** |  |  |
| Length | 335 aa | 354 aa |
| Molecular weight | 38 kDa | 40 kDa |
| pI | 8.5 | 8.6 |
