# Supplementary material for: A mitochondrial rRNA dimethyladenosine methyltransferase in Arabidopsis
Source: Plant J. 2010 Feb;61(4):558–69. doi: 10.1111/j.1365-313X.2009.04079.x (PMC2860759; doi:10.1111/j.1365-313X.2009.04079.x)
Supplement: Supplementary file 7 [file tpj0061-0558-SD7.doc]

**Supplementary Table S2: Analyzed promoter sequences.** Initiating nucleotides are underlined; typical core promoter sequence motifs are written bold, other important sequence elements are shaded.

| Gene | Promoter | Sequence |
| --- | --- | --- |
| *rrn18* | P*rrn18*-156 | TAGAATAATA**CGTA**TATAATCAGAA |
| *atp6-1* | P*atp6-1*-200 | GCCAATAATA**CGTA**TATAAGAAGAG |
| *atp9* | P*atp9*-295 | CTGGTGCTCT**CGTA**TATAAGAGAAG |
| *cox2* | P*cox2*-210 | ATGTTGGTTT**CGTA**TATAAGAAGAC |
| *tRNA-fMet* | P*trnM*-98 | TTTGAAATAT**CGTA**AGAGAAGAAGG |
| *rrn26* | P*rrn26*-893 | CTATCAATTT**CATA**AGAGAAGAAAG |
| *atp9* | P*atp9*-239 | CTATCAATTT**CATA**AGAGAAGACGA |
| *atp6-1* | P*atp6-1*-156 | CTATCAATCT**CATA**AGAGAAGAAAT |
| *atp8* | P*atp8*-157 | CTATCAATCT**CATA**AGAGAAGAAAT |
| *rrn18* | P*rrn18*-69 | AGTGGAATTG**AATA**AGAGAAGAAAG |
| *atp8* | P*atp8*-228/226 | CATACCATAA**CATA**TATAGAATCGA |
| *atp6-1* | P*atp6-1*-916/913 | AGCCCTTTAT**ATTA**TATAATAAAGC |
| *cox2* | P*cox2*-481 | ATGAATATTC**ATTA**GATAATAGATT |
| *rps3* | P*rps3*-1133 | TAGAAAAAATT**ATTA**GTAATACGTA |
| *rps3* | P*rps3*-1053 | TTTTTTATTT**GGTA**GGTAACATCGC |
| *atp9* | P*atp9*-487 | ATGTCTTATT**GGTA**TGTGATACAAG |
| *atp9* | P*atp9*-652 | AGAAGATTGA**AGTA**AGGAGCAGGTT |
| *atp6-2* | P*atp6-2*-436 | TCTTGAATTA**AGTA**TATAGAAAAGA |
| *atp6-2* | P*atp6-2*-507 | GATAAATTA**AGTA**TAGTAATAAGAA |
| *tRNA-fMet* | P*trnM*-574/573 | CTAATTTATATAAAAAAGACCGGGA |
