# Supplementary material for: A mitochondrial rRNA dimethyladenosine methyltransferase in Arabidopsis
Source: Plant J. 2010 Feb;61(4):558–69. doi: 10.1111/j.1365-313X.2009.04079.x (PMC2860759; doi:10.1111/j.1365-313X.2009.04079.x)
Supplement: Supplementary file 8 [file tpj0061-0558-SD8.doc]

**Supplementary Figure S5. Run-off transcription from the mitochondrial *atp6-1*, *atp8* and *rrn18* upstream regions by RPOTm and RPOTmp.** (**A**) RPOTm and RPOTmp were assayed for promoter-specific transcription from plasmids pKL23-*atp6-1*, pKL23-*atp8* and pKL23-*rrn18* provided as supercoiled (lanes labelled ccc) or linearized DNA templates (lin). *In vitro* transcription reactions were run in the presence or absence of Dim1B (2) or Dim1A (1) as indicated. Labelled RNA products were separated in a denaturing polyacrylamide gel alongside an RNA size marker; sizes are given in nucleotides (indicated on the left). Major discrete RNA products obtained with supercoiled templates are indicated by arrows and, according to our previous *in vitro* transcription analyses (Kü*hn et a*l. 2007), attributed to transcription initiation at P*atp6-1*-200 followed by termination at terminator sequences *hisa* (signals labelled P-200-*hisa*) or *thra* (signal P-200-*thra*), initiation at Patp8-228/226 and termination at *hisa* or *thra*, and initiation at P*rrn18*-156 and termination at *hisa* or *thra*. Extended expositions of gels did not reveal additional bands. (**B**) DNA templates were constructed by inserting *Arabidopsis* mitochondrial promoter regions (grey bars) into pKL23 (Liere and Maliga 1999) upstream of the two bacterial ρ-independent terminator sequences *hisa* (Barnes and Tuley 1983) and *thra* (Gardner 1982). When providing a supercoiled pKL23 derivative as template, transcription initiated at the introduced promoters is terminated at *hisa* and/or *thra*, thereby generating RNA products of distinct lengths. Transcripts expected from initiation at mitochondrial promoters P*atp6-1*-156, P*atp6-1*-200, Patp8-157, Patp8-228/226, P*rrn18*-69 or P*rrn18*-156 (bent arrows) and termination at *hisa* or *thra* (triangles) or at the restriction sites *Eco*RI (E) used for linearization of pKL23-*atp6-1* or *Xho*I (X) used for pKL23-*atp8* and pKL23-*rrn18* linearization are indicated by horizontal black arrows labelled with the respective RNA lengths. Minor signals indicated by grey arrows labelled with asterisks may be due to differently migrating major products.

**Barnes, W.M. and Tuley, E.** (1983) DNA sequence changes of mutations in the histidine operon control region that decrease attenuation. *J Mol Biol*, **165**, 443-459.

**Gardner, J.F.** (1982) Initiation, pausing, and termination of transcription in the threonine operon regulatory region of Escherichia coli. *J Biol Chem*, **257**, 3896-3904.

**Kühn, K., Bohne, A.-V., Liere, K., Weihe, A. and Börner, T.** (2007) Arabidopsis single-polypeptide RNA polymerases: accurate in vitro transcription of organellar genes. *Plant Cell*, **19**, 959–971.

**Liere, K. and Maliga, P.** (1999) In vitro characterization of the tobacco rpoB promoter reveals a core sequence motif conserved between phage-type plastid and plant mitochondrial promoters. *EMBO J*, **18**, 249-257.
